# Surgery and Acute Stress Decrease NRF2 mRNA Expression and Promote Iron Metabolism Alteration, Oxidative Stress, and Inflammatory Gene Expression in the Liver of Prehypertensive Rats

**DOI:** 10.33549/physiolres.935716

**Published:** 2025-12-01

**Authors:** Michal KLUKNAVSKY, Peter BALIS, Andrea MICUROVA, Martin SKRATEK, Jan MANKA, Iveta BERNATOVA

**Affiliations:** 1 Centre of Experimental Medicine, Slovak Academy of Sciences, Institute of Normal and Pathological Physiology, Bratislava, Slovakia; 2 Institute of Measurement Science, Slovak Academy of Sciences, Bratislava, Slovakia

**Keywords:** Non-hepatic surgery, Air-jet stress, Gene expression, Magnetic properties, Oxidative damage

## Abstract

This study investigated how non-hepatic surgery and subsequent acute stress affect iron distribution, redox state, antioxidant defence, and inflammation-related gene expressions and iron metabolism in the liver of borderline hypertensive rats. We used air-jet stress as a model of acute psychological stress (3 sessions of 5 sec. air-jet) applied approximately 22 hours post-surgery (carotid artery and jugular vein cannulation). Both the surgery (Su) and post-surgical stress (Su+Str) increased corticosterone and reduced iron concentrations in plasma, while increasing remanent magnetisation (*M_r_*) and coercivity (*H_c_*) in whole blood. In the liver, Su and Su+Str reduced mRNA expressions of genes encoding NFR2 and GPX4 proteins (*Nfe2l2* and *Gpx4*, respectively), and induced a significant increase in hepatic conjugated dienes, proinflammatory factors (*Il1b*) and iron-regulating genes mRNA (*Hmox1, Fpn1, Fth1, Hamp, Tfr1*), despite elevated *Hmox1* and *Sod1* mRNA expressions. In addition, hepatic *M_r_* and *H_c_* after Su and Su+Str were elevated, suggesting a qualitative change of iron-containing substances in circulation and liver tissue. In addition, in the Su+Str group, the elevated saturation magnetisation (*M_s_*) is indicative of elevated total iron content. These findings suggest that a mild non-hepatic surgery may reduce hepatic mRNA expression of NRF2 and GPX4, which was associated with oxidative tissue damage accompanied by qualitative alterations in cellular iron, indicating a pro-ferroptotic state that, together with enhanced inflammation, may contribute to post-surgical liver injury. Additionally, the combination of surgery and acute post-surgical stress led to tissue iron accumulation, which may contribute to liver damage.

## Introduction

Minor surgical interventions trigger a complex physiological response, known as the surgical stress response, characterized by neuroendocrine activation, inflammation, and metabolic alterations aimed at preserving homeostasis and facilitating tissue repair [1]. Patients with elevated blood pressure (BP) may exhibit a higher susceptibility to post-surgery complications [2]. Thus, we used adult borderline hypertensive rats (BHR), reflecting a human population with prehypertension, which affects 25–50 % of adults worldwide [3]. BHR exhibit heightened sympathetic tone and enhanced sensitivity to external stress, which makes them a relevant model to test the effects of surgical and acute stress [4,5].

During the acute stress response, significant changes occur in iron metabolism, which was confirmed by multiple experimental studies. Acute psychological stress led to increased hepcidin protein levels, reduced ferroportin expression in the liver, and was accompanied by elevated iron accumulation and oxidative damage [6, 7]. Similar iron accumulation in the liver was noted in a study on rodents with social defeat stress, where the authors also noted increased plasma levels of ferritin and hepcidin, accompanied by a decrease in plasma iron [8].

Clinical and preclinical studies show that surgical trauma, sepsis, and psychological stress all induce significant disturbances in iron metabolism. Increased serum ferritin and hepcidin levels following surgery or trauma served as part of a protective host response to limit pathogen access to iron [9,10]. Elevated hepcidin levels, driven by systemic inflammation, are associated with reduced circulating iron and play a key role in the development of anemia of inflammation frequently observed after surgery [11]. However, such redistribution can also promote tissue damage through redox-active iron and reactive oxygen species generation. In patients with cardiometabolic risk, this imbalance may increase susceptibility to hepatic injury and metabolic complications [12].

Systemic and oxidative stress modulate the antioxidant defense system, where nuclear factor erythroid 2-related factor 2 (NRF2) acts as its central transcriptional regulator. Upon oxidative challenge, NRF2 translocates to the nucleus, and binds to antioxidant response elements within the promoters of target genes [12]. After binding to the promoter, NRF2 regulates a wide array of cytoprotective genes and genes involved in antioxidant defence [13]. NRF2 also influences iron metabolism by regulating the expression of genes involved in iron sequestration (notably *Fth1*, *Tfr1*) and export (*Fpn1*), thereby limiting the pool of redox-active iron associated with oxidative damage. This function is particularly relevant in the context of ferroptosis, a form of regulated cell death dependent on iron and lipid peroxidation [14].

Our study aimed to investigate the impact of surgical trauma and post-surgery acute stress on systemic and hepatic iron metabolism, oxidative stress, and related gene expression in an experimental model of prehypertension. Specifically, we aim to determine: a) the changes in plasma and hepatic iron levels following surgery and acute air-jet stress, b) alterations in hepatic magnetic properties, indicative of iron content and form, c) the hepatic markers of oxidative stress and inflammation and d) expression levels of genes involved in iron metabolism, oxidative stress response, inflammation, and ferroptosis. We hypothesized that i) surgical intervention will lead to redistribution of iron from plasma to the liver, accompanied by upregulation of inflammatory mediators and oxidative damage to the liver, ii) acute stress following surgery will exacerbate hepatic iron accumulation, oxidative stress, and inflammatory responses, and iii) these changes will be reflected in the altered expression of genes regulating iron homeostasis, antioxidant defense system, inflammation and ferroptosis signaling pathways.

## Materials and Methods

### Animals and treatment

All procedures used in this study were approved by the Ethics Committee of the Centre of Experimental Medicine, Slovak Academy of Sciences, Bratislava, Slovakia and by the State Veterinary and Food Administration of the Slovak Republic, protocol code Ro-2654-3/2021-220.

Sixteen-week-old BHR males, the offspring of SHR dams and WKY sires, were used in this study. All rats were born in our certified animal facility (Institute of Normal and Pathological Physiology SAS) in order to maintain the same environmental background for all animals. The BHR were housed in a 12 h light/12 h dark cycle at constant humidity (45–65 %) and temperature (20–22 °C) and had free access to standard laboratory rat chow (Altromin 1324P, Altromin International, Lage, Germany) and tap water *ad libitum*. Rats (n=23) were divided into a control group (Cont, n=7), a group that underwent surgery (Su, n=7), and a group that underwent surgery and was exposed to post-surgical repeated acute air-jet stress (Su+Str, n=9). All BHR were housed under standard laboratory conditions in groups of 2–3 per cage (39×23.3×23 cm).

All rats designated for surgery (Su and Su+Str group) were surgically implanted with two catheters under general anesthesia (2.5–3.5 % isoflurane) one day before the experiment, as described previously [15]. Catheters were inserted into the left carotid artery and the jugular vein. The catheters were exteriorized in the interscapular region, and animals were allowed to recover from anesthesia for approximately 20–22 hours. Acute stress was induced by a 5-second pulse of compressed air directed at the rat’s face (air-jet). The detailed procedure for air-jet stress exposure, post-surgery care and experimental setup was described in a previous study [16].

At the end of the experiment, blood samples were collected from the trunk of control rats. In the surgery-exposed groups, blood samples (~250 μl) were obtained from the carotid artery by catheter into Li-heparinized tubes. From the obtained blood, 33 μl was used for the assessment of the blood’s magnetic properties. The remaining blood from all animals was centrifuged (850 g, 10 min, 4 °C) to obtain blood plasma. After collecting blood samples, all rats were briefly anesthetized with CO_2_ and decapitated. The liver and adrenal glands (AG), cleaned of surrounding fat and connective tissue, were weighed for biometric analysis and dissected for magnetometric, biochemical and molecular analysis. Liver samples were collected from the central region of the left lateral lobe, which physiologically contains the highest concentration of iron [17]. Subsequently, the obtained liver, blood and plasma samples were snap-frozen in liquid nitrogen and stored at −80°C until further processing.

### Systolic blood pressure and heart rate determination

In all groups, systolic blood pressure (BP) and heart rate (HR) were measured before surgery non-invasively using tail-cuff plethysmography with the CODA system (Kent Scientific Corporation, Torrington, CT, USA) between 11:00 a.m. and 12:00 p.m., as previously described [18]. To minimize nonspecific stress effects, rats were habituated to the procedure in three separate handling sessions before the experiment. Systolic BP is expressed in millimeters of mercury (mmHg), and heart rate in beats per minute (BPM).

### Measurement of biometric parameters

The final body weight (BW), liver and adrenal glands (AG) weight of each rat were determined on the day of the experiment. The Liver/BW ratio expresses the degree of hyper-/hypotrophy of the liver relative to BW. The AG/BW ratio expresses the degree of hyper-/hypotrophy of the AG relative to BW.

### Determination of total iron and divalent iron in plasma

Plasma levels of total iron, ferrous iron (Fe^2+^) and ferric iron (Fe^3+^) were determined using an Iron Assay Kit (ab83366, Abcam) according to the manufacturer’s protocol (ab83366, Abcam, Cambridge, UK). A 50 μL aliquot of the plasma sample was diluted 1:1 with Iron Assay Buffer, resulting in a final volume of 100 μL. Plasma samples diluted with Iron Assay Buffer were added to 96-well plate. The free Fe^2+^ reacted with the iron probe, forming a stable colored complex. The free Fe^3+^ was subsequently reduced by the iron reducer to Fe^2+^ for total iron determination. The samples were measured with a microplate reader (BioTek 800 TS, Tianjin, China) at 593 nm.

### Assessment of plasma corticosterone levels

Plasma corticosterone (Cort) levels were measured using a commercial colorimetric ELISA kit (ab108821, Abcam). Cort was measured in 25 microliters of plasma that was diluted 4-fold with distilled water. All reagents were equilibrated to room temperature and prepared according to the manufacturer’s protocol. All standards, controls, and samples were assayed in duplicates and were measured with a microplate reader (BioTek 800 TS, Ltd., Tianjin, China) at 450 nm.

### Determination of magnetic parameters in blood and liver

Biogenic iron content was determined in the liver and blood samples using a Quantum Design MPMS-XL 7AC SQUID magnetometer with reciprocating sample operation and 10^−11^ Am^2^ sensitivity, as previously described [19].

Before analysis, liver samples were allowed to thaw before being cut to a uniform shape with a diameter of ~4.5 mm using a cylindrical instrument. The samples were subsequently dried under vacuum conditions, weighed, and inserted into plastic measurement tubes. Blood sample (33 μL) was pipetted onto a pre-weighed strip of standard office paper (80 g/m^2^), measuring 18 cm in length and 6 mm in width. The sample was then air-dried at room temperature for 24 hours, weighed, and placed inside a plastic measuring straw.

Saturation magnetization values (*M_s_*) refer to the maximum magnetization induced by an external magnetic field in the material and are used to determine the relative content of magnetic compounds in the liver, with iron being the dominant component. Remanent magnetization (*M_r_*) refers to the amount of magnetism retained in a substance after the removal of a magnetic field sufficient to reach *M_s_*. The magnetic field strength, needed to reduce this remanent magnetization to zero, is known as magnetic coercivity (*H_c_*) [19]. Parameters *M_r_* and *H_c_* are parameters which depend on the size and chemical moiety of the iron-containing substance. *H_c_* is expressed as Oersted units (Oe). *M_s_* and *M_r_* are express*ed* as electromagnetic units of magnetic moment of dried sample weight (emu/g).

### Measurement of conjugated dienes content in the liver

Conjugated dienes (CD), a marker of lipid peroxidation and oxidative damage, were quantified in 10 % (w:v) tissue homogenates of the liver. The detailed procedure for CD processing and isolation has been previously described [19]. The absorbance of the samples was measured at 233 nm, and the results were calculated using an extinction coefficient of 26,000 mol^−1^·L·cm^−1^. The final results were expressed as nanomoles of CD per gram of tissue (nmol/g).

### Gene expression analysis in the liver

The gene expression levels of nuclear factor erythroid 2-related factor 2 (*Nfe2l2* encoding NRF2 protein), superoxide dismutase 1 (*Sod1* encoding SOD1 protein), heme oxygenase 1 (*Hmox1* encoding HO-1 protein), glutathione peroxidase 4 (*Gpx4* encoding GPX4 protein), peroxisome proliferator-activated receptor alpha and gamma (*Ppara* and *Pparg* encoding PPAR-α and PPAR-γ protein, respectively), tumor necrosis factor alpha (*Tnf* encoding TNF-α protein), interleukin 1 beta (*Il1b* encoding (IL-1β protein), ferroportin (*Fpn1*/*Slc40a1* encoding FPN1 protein), transferrin receptor 1 (*Tfr1* encoding TfR1 protein), iron regulatory protein 1 (*Irp1*/*Acon1* encoding IRP1 protein), divalent metal ion transporter 1 (*Dmt1/Slc11a2* encoding DMT1 protein), hepcidin (*Hamp* encoding hepcidin protein) and ferritin heavy chain (*Fth1* encoding FTH protein) were determined in the liver by using real-time quantitative polymerase chain reaction (RT-qPCR). 60S ribosomal protein L10a (*Rpl10a* encoding RPL10A protein) was used as housekeeping gene.

The total RNA was isolated using the PureZOL™ RNA Isolation Reagent (Bio-Rad, Hercules, CA, USA), according to the manufacturer’s protocols. The amount and purity of total isolated RNA were spectrophotometrically quantified at 260/280 nm and 260/230 nm while using a NanoDrop spectrophotometer (Thermo Scientific, Waltham, MA, USA). Reverse transcription was performed using 1 μg of total RNA from each sample using Eppendorf Mastercycler (Eppendorf, Hamburg, Germany) and an iScript-Reverse Transcription Supermix (Bio-Rad, Hercules, CA, USA), according to the manufacturer’s protocols. Gene-specific primers were designed using the PubMed program (Primer-BLAST) and database (Gene). The DNA sequences and melting temperature (T_m_) of the used primers, the size of the amplicons in base pairs (bp), and the reference numbers of the templates are described in Table 1.

The precise composition of the PCR mixture, the amount and dilution of the cDNA template, thermal cycling parameters of the PCR and data analysis have been described in detail in a previous study [20].

### Statistical analysis

All data in the above study were analyzed by one-way analysis of Variance (ANOVA and Bonferroni post-hoc test). The normality of the data distribution was tested using the Shapiro-Wilk test. The homogeneity of data was tested by Levene’s test. In the case of a significant Levene’s test, we used Welch’s ANOVA for data analysis, followed by the Games–Howell post-hoc test. Correlations between variables were analyzed using Pearson’s correlation coefficient (r). The values were considered to differ significantly when p<0.05. The results are presented as mean±standard error of means (SEM). The GraphPad Prism v7.02 software (GraphPad Software, Inc., San Diego, CA, USA) and Statistica v13.5 (StatSoft Europe, Hamburg, Germany) were used for the statistical analyses. Unless otherwise stated, all statistical analyses were performed on all animals within the individual experimental groups.

## Results

### Biometric and hemodynamic parameters

**Fig. 1:**
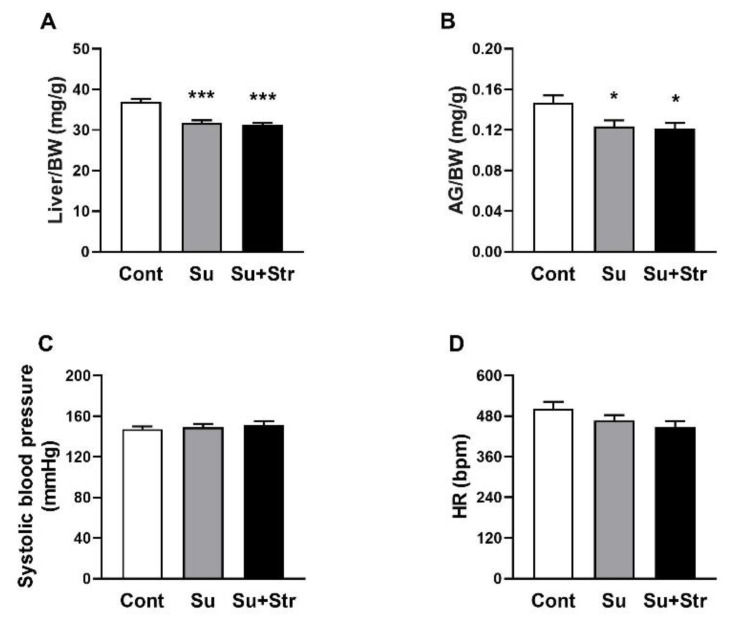
The effect of surgery and acute post-surgical stress on Liver/BW ratio (**A**), AG/BW ratio (**B**), systolic blood pressure (**C**) and HR values (**D**). Values represent the mean±SEM. n = 7 – 9/group, * p<0.05, ** p<0.01, *** p<0.001 vs. Cont group. Abbreviations: AG – adrenal glands, bpm – beats per minute, BW – body weight, HR – heart rate, Cont – Control group, Su – surgery-exposed group, Su+Str – surgery+acute stress-exposed group.

On the day before surgery, the body weights of rats in the Cont group (374.6±4.2 g) were comparable to those in the Su (363.4±3.9 g) and Su+Str (354.8±7.6 g) groups. After the surgical procedure, BW of rats in Su (360.1±4.5 g) and Su+Str (353.0±7.7 g) groups remained unchanged. Cont rats had a significantly (p<0.001) higher Liver/BW value (36.97±0.76 mg/g) compared to surgery-exposed rats in Su (31.76±0.63 mg/g) and Su+Str (31.28±0.51 mg/g) groups (Fig. 1A). Statistical analysis confirmed significantly (p<0.05) reduced AG/BW values in Su (0.124±0.006 mg/g) and Su+Str (0.121±0.005 mg/g) groups compared to the Cont group (0.147±0.019 mg/g) (Fig 1B).

With respect to hemodynamic parameters, all rats assigned to the surgical groups (Su and Su+Str) exhibited similar values of systolic BP (149±3 mmHg and 151±4 mmHg, respectively) and HR (467±16 bpm and 448±18 bpm, respectively) on the day before surgery, when compared with rats assigned to the Cont group (147±3 mmHg and 502±21 bpm) (Fig. 1C,D). All these findings suggest surgery-induced stress associated with AG hypotrophy and liver hypotrophy.

### Plasma levels of corticosterone, total iron and divalent iron in plasma

**Fig. 2:**
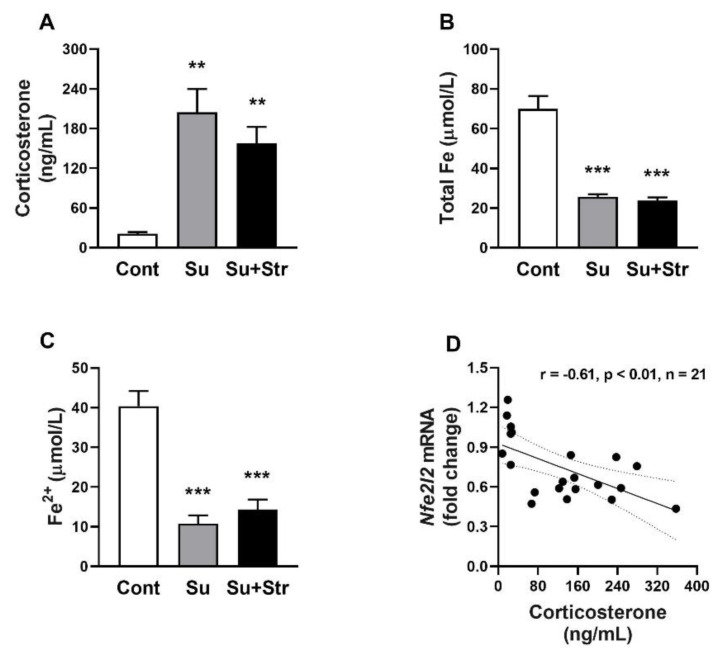
The effect of surgery and acute post-surgical stress on plasma levels of corticosterone (**A**), total Fe (**B**), Fe^2+^(**C**) and correlation between plasma corticosterone levels and liver expression of *Nfe2l2* gene (**D**). Values represent the mean±SEM. Cont group. n = 7/group, ** p<0.01, *** p<0.001 vs. Abbreviations: *Nfe2l2* – nuclear factor erythroid 2-related factor 2, Cont – Control group, Su – surgery-exposed group, Su+Str – surgery+acute stress-exposed group.

Both surgery-exposed groups, Su (204.61±35.39 ng/mL) and Su+Str (157.83±24.83 ng/mL), had significantly (p<0.01) elevated corticosterone levels compared to the Cont rats (21.18±2.54 ng/mL) (Fig. 2A). Cont rats had a significantly (p<0.001) higher total Fe levels (69.98±6.49 μmol/L) compared to Su (25.72±1.21 μmol/L and Su+Str (23.83±1.53 μmol/L) groups (Fig. 2B). Statistical analysis confirmed significantly (p<0.001) reduced levels of Fe^2+^ in Su (40.0±3.79 μmol/L) and Su+Str (10.71±2.16 μmol/L) groups compared to the Cont group (14.34±2.49 μmol/L) (Fig. 2C). All these findings suggest surgery-induced stress accompanied by a drop in iron content and an increase in circulating corticosterone levels. Data analysis revealed a negative correlation between corticosterone levels and *Nfe2l2* gene expression (Fig. 1D).

### Magnetic properties of the liver and blood

**Fig. 3:**
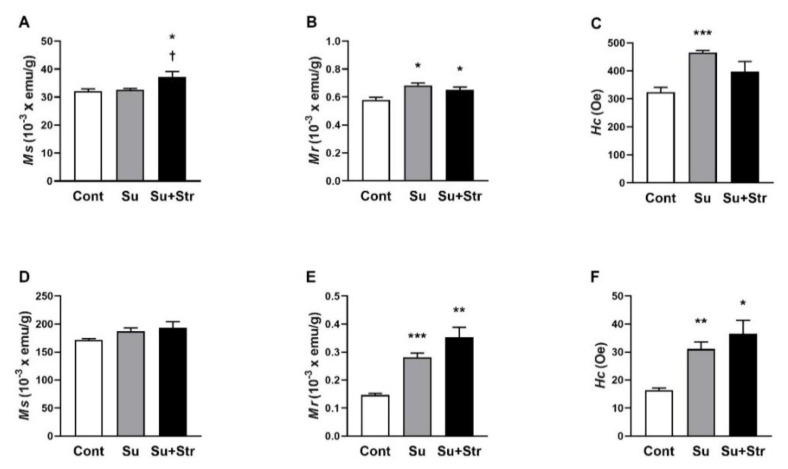
The effect of surgery and acute post-surgical stress on magnetic parameters *M_s_*, *M_r_* and *H_c_* in the liver (**A–C**) and blood (**D–E**). Values represent the mean±SEM. n = 7/group, * p<0.05, ** p<0.01, *** p<0.001 vs. Cont group; † p<0.05 vs. Su group. Abbreviations: *Ms* – saturation magnetisation, *Mr* – remanent magnetisation, *Hc* – coercivity, Cont – Control group, HR – heart rate, Su – surgery-exposed group, Su+Str – surgery+acute stress-exposed group.

In the liver, Su+Str group showed a significant (p<0.05) increase in *M_s_* values compared to the remaining groups (Fig. 3A). Cont rats exhibited significantly (p<0.05) lower *M_r_* values (0.579±0.020) × 10^−3^ emu/g compared to both the Su (0.684±0.017) × 10^−3^ emu/g and Su+Str groups (0.675±0.024) × 10^−3^ emu/g (Fig. 3B). Statistical analysis confirmed significantly (p<0.05) elevated *H_c_* values in the liver of Su group (465.60±6.98 Oe) with a similar insignificant trend in Su+Str group (399.38±32.73 Oe) compared to the Cont group (323.43±17.77 Oe) (Fig. 3C).

In the blood, there were no significant differences in *M_s_* values between the surgery-exposed and Cont groups (Fig. 3D). Significantly (p<0.001 and p<0.01) increased *M_r_* values were found in Su group (0.281±0.015) × 10^−3^ emu/g and Su+Str group (0.353±0.035) × 10^−3^ emu/g compared to Cont group (0.146±0.006) × 10^−3^ emu/g (Fig. 3E). A similarly significant (p<0.01 and p<0.05) increase in *Hc* values was observed in the Su group (31.04±2.54 Oe) and the Su+Str group (36.57±4.76 Oe) compared to the Cont group (15.93±0.56 Oe) (Fig. 3F).

Our results indicate qualitative changes in the iron present in the blood and liver tissue associated with the surgical procedure.

### Analysis of gene expression

**Fig. 4:**
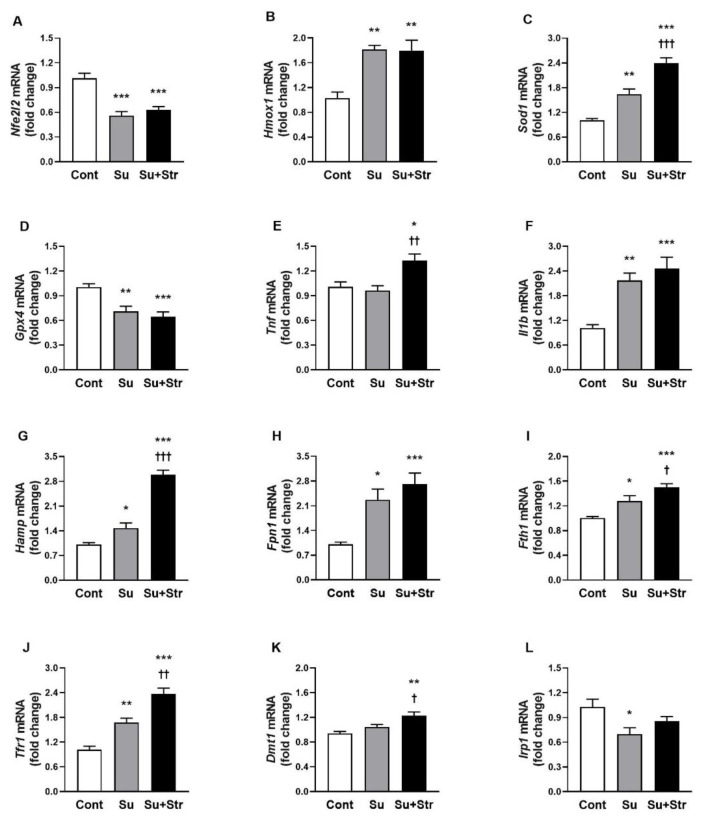
The effect of surgery and acute post-surgical stress on gene expression. Values represent the mean ± SEM. Cont group. n = 7–9/group, * p < 0.05, ** p <0.01, *** p < 0.001 vs. Cont group; † p <0.05, †† p < 0.01, ††† p < 0.001 vs. Su group. Abbreviations: Cont – Control group, HR – heart rate, Su – surgery-exposed group, Su+Str – surgery+acute stress-exposed group. Gene names are explained in the legend to Table 1.

Gene expression analysis revealed a significant (p<0.001) decrease in *Nfe2l2* gene, in both surgery-exposed groups compared to the Cont group (Fig. 4A). Despite the reduced expression of *Nfe2l2*, a significant (p<0.01) upregulation of NRF2-regulated antioxidant defence genes *Hmox1* and *Sod1* was observed in both Su and Su+Str groups (Fig. 4B,C), while *Gpx4* expression showed an opposite significant (p<0.01) change compared to Cont animals (Fig. 4D). The interaction between surgery and post-surgical stress led to a significant (p<0.001) increase of *Sod1* expression in the Su+Str group compared to the Su group (Fig. 4C). Regarding pro-inflammatory factors, the expression of *Il-1β* was significantly (p<0.01) increased in both Su and Su+Str groups (Fig. 4F). Additionally, the combined Su+Str group exhibited a significant upregulation of the pro-inflammatory factor *Tnf* compared to the Cont group (Fig. 4E). Analysis of iron metabolism–related genes revealed an increased expression of *Hamp*, *Fpn1*, *Fth1* and *Tfr1* in both surgery-exposed groups (Fig. 4G–4J). The interaction between surgery and acute post-surgical stress also led to increased expression of the iron-regulatory gene *Dmt1* compared to the Cont group (Fig. 4K), as well as a marked upregulation of *Hamp*, *Fth1*, and *Tfr1* genes relative to the Su group (Fig. 4G, 4I, 4J). For *Irp1* gene, a significant (p<0.05) downregulation was observed in the Su group and a non-significant decrease in the Su+Str group (Fig. 4L).

### Conjugated diene levels and their correlations with antioxidant genes expression in the liver

**Fig. 5:**
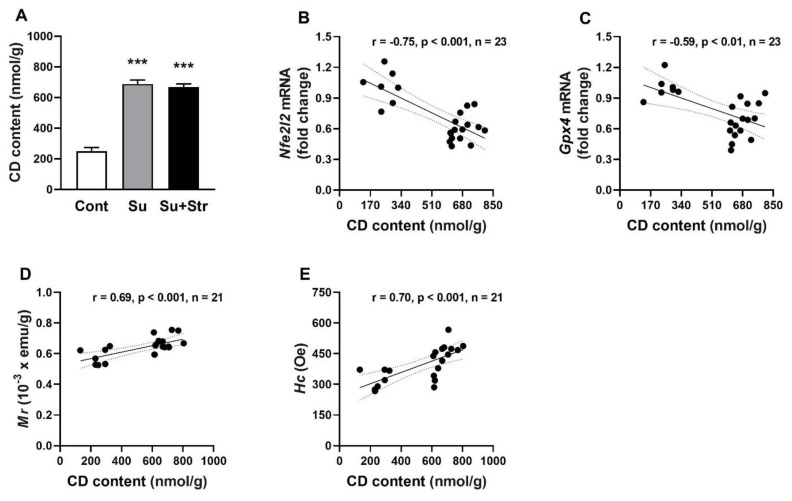
The effect of surgery and acute post-surgical stress on CD content (**A**) and correlation between CD content and gene expression of *Nfe2l2* (**B**), *Gpx4* (**C**), *M_r_* values (**D**), *H_c_* values (**E**) in the liver. Values represent the mean ± SEM. n = 7 – 9/group, ** p < 0.01, *** p <0.001 vs. Cont group. Abbreviations: *Nfe2l2* – nuclear factor erythroid 2-related factor 2, CD – conjugated dienes, *Gpx4* – glutathione peroxidase 4, *Mr* – remanent magnetisation, *Hc* – coercivity, Cont – Control group, HR – heart rate, Su – surgery-exposed group, Su+Str – surgery+acute stress-exposed group.

Both surgery-exposed groups, Su and Su+Str, had similar (688.40±25.02 nmol/g and 669.08±19.59 nmol/g) and significantly (p<0.001) increased CD content compared to Cont rats (250.01±23.98 nmol/g) (Fig. 5A). Statistical analysis revealed a strong correlation between CD content and the expression of *Nfe2l2* and *Gpx4* genes, as well as *M_r_ and H_c_* (Fig. 5B–5E).

## Discussion

This study provides new insights into how even mild surgical intervention followed by acute stress affects oxidative stress, inflammatory signalling and iron metabolism in the liver of young adult BHR rats. While the stress response to surgery and psychological burden has been extensively studied in clinical and preclinical models, our approach integrates magnetometry, hepatic gene expression, and markers of oxidative damage and inflammation to evaluate the combined impact of surgery and acute psychoemotional stress in rats with prehypertension. The key findings of our study can be summarized as follows: i) Minor surgical intervention alone significantly altered systemic and hepatic iron homeostasis and induced hepatic inflammation, ii) The addition of acute air-jet stress, approximately one day post-surgery, further intensified these effects, leading to elevated hepatic iron content associated with increased magnetic parameters (*M_s_*, *M_r_* and *H_c_*), upregulation of multiple genes involved in iron metabolism, antioxidant defense and inflammation, and enhanced oxidative lipid damage.

Analysis of the liver-to-body weight ratio (Liver/BW) revealed reduced relative hepatic mass in both surgery-exposed groups. Given that the overall body weight of the rats showed only a mild and non-significant decrease compared with preoperative values, we suggest that the observed hepatic hypotrophy was primarily induced by surgical stress. Surgical stress may induce the depletion of glycogen and water from the liver, which may account for the observed acute reduction in Liver/BW ratio [21, 22].

We observed that surgery-induced stress was associated with a decrease in plasma iron. These findings are similar to clinical findings which showed that trauma, surgical procedure and critical illness have been associated with reduced iron content in circulation [23–25]. Surgery-induced hypoferremia was associated with elevated hepcidin levels and may serve to restrict iron-dependent microbial growth. However, it simultaneously contributes to hypoferremia, oxidative damage, and impaired erythropoiesis [9]. However, decreased plasma iron levels detected by biochemical analysis were not accompanied by reduced *M_s_* values in whole blood, which supposedly reflects quantitative alterations in iron. This apparent discrepancy likely reflects that the majority of iron in the blood is contained within red blood cells, whereas only a small fraction is bound to transport proteins or exists as free iron in the plasma [26]. Therefore, decreased iron levels in plasma in our study may not be reflected in changes in total blood iron content measured by magnetometry. Although the effects of non-hepatic surgery on systemic iron levels are well recognized in human, there is less information on its impact on hepatic iron metabolism. In rodent models, acute psychological stress was associated with a decrease in circulating iron and liver iron accumulation, oxidative damage and reduced glutathione levels [6–8].

We determined the magnetic properties of hepatic iron using SQUID magnetometry to analyze quantitative and qualitative changes in magnetic forms of iron [19]. In both surgery-exposed groups, we found increased values of *H_c_* and *M_r_* in the liver with similar significant changes in whole blood. These findings suggest a shift in the chemical form of iron toward more oxidized and magnetically harder species-likely ferrihydrite or partially oxidized magnetite [27]. Both surgery-exposed groups had considerably increased oxidative damage to lipids in the liver. The comparable level of oxidative damage, which strongly correlated with the qualitative magnetic parameters (*H_c_* and *M_r_*) in the liver of both surgery-exposed groups, suggests that oxidative damage was associated with qualitative alterations of intracellular iron-containing substances. The previous study showed that alterations in the structural organization of ferritin-bound iron may render stored redox-active iron, explaining the increase in oxidative damage [28]. Oxidative stress–induced damage of ferritin structure may also account for the qualitative iron changes detected by magnetometry, reflecting an increased pool of labile iron associated with the progressive oxidative damage in the liver [29]. Through Fenton chemistry, iron catalyzes the formation of hydroxyl radicals, contributing to lipid peroxidation and hepatocellular injury [30]. In addition, oxidative damage to liver lipids could be caused by reduced *Gpx4* expression [31].

Iron-dependent oxidative damage is also tightly linked with other genes involved in iron metabolism. In our study, hepatic expression of *Hamp*, *Tfr1*, *Fpn1* and *Fth1* was significantly increased in both surgery-exposed groups, indicating augmented iron storage (*Fth1*), elevated cellular influx (*Tfr1*) and reduced efflux (*Hamp, Fpn1*) of iron in the liver due to non-hepatic surgical procedure. Several studies have reported time-dependent alterations in the hepatic expression of *Hamp*, *Tfr1*, *Fpn1*, and *Fth1* genes in rodents, depending on the time elapsed since the surgical procedure and the specific model of liver injury employed [32,33]. In studies where turpentine oil injections led to non-hepatic tissue damage in rodents, significant changes in the expression of the *Hamp*, *Tfr1*, *Fpn1*, and *Fth1* genes were observed. Similarly to the studies with induced liver damage, the changes in gene expression varied according to the post-surgery time of gene analysis [32, 34]. The increased expression of *Fth1* in both surgery-exposed groups may reflect an attempt to reverse ferritin damage associated with the release of free iron into hepatocytes. In addition, increased expression of *Dmt1* gene together with elevated *M_s_* values in the liver Su+Str group may indicate increased hepatic ferrous iron accumulation after post-surgical stress that can further accelerate damage to the hepatocytes.

In our study, elevated lipid damage was accompanied by downregulated expression of *Nfe2l2* and *Gpx4* genes in both surgery-exposed groups. The repression of *Gpx4* and *Nfe2l2* in the liver suggests a prooxidative and pro-ferroptotic state, which was found previously [14]. Ferroptosis represents a form of iron-dependent cell death characterized by increased lipid peroxidation and GPX4 suppression, and it is increasingly recognized in liver diseases, including NAFLD, ischemia-reperfusion injury, and alcohol-induced liver damage [35]. Although we did not directly confirm ferroptotic cell death (e.g., through ferrostatin-1 rescue), the gene expression pattern (reduced *Nfe2l2* and *Gpx4* expressions) and magnetometric findings are consistent with ferroptosis-prone conditions.

Surgery-induced stress, associated with a decrease in mRNA of NRF2, is consistent with previous studies in which mRNA and protein levels of NRF2 were reduced in rats exposed to stress [36,37].

Moreover, reduced *Nfe2l2* expression may have also been a consequence of high corticosterone levels. In our study, surgery-induced stress was manifested by ~10-fold increased plasma corticosterone in both Su and Su+Str groups. The hypothesis of corticosterone-mediated repression of *Nfe2l2* expression in the liver was indirectly confirmed by a negative correlation between *Nfe2l2* expression and plasma corticosterone levels. This is in agreement with the studies that found that glucocorticoids can repress the expression of NRF2 and several NRF2-target genes by binding the glucocorticoid receptor to the ARE, in response to elevated glucocorticoids [38]. In addition, glucocorticoids can also suppress *Nfe2l2* expression by antagonizing transcription factors such as NF-κB and AP-1, which are known positive regulators of *Nfe2l2* expression [39,40]. Specifically in the liver, acutely increased glucocorticoid levels were accompanied by attenuated *Nfe2l2* mRNA and NRF2 protein levels, impairing the antioxidant response and increasing susceptibility to oxidative damage [36].

Our study also highlights the modulatory effect of acute post-surgical stress on liver inflammation. The surgical procedure alone induced an increase in hepatic *Hamp* expression, accompanied by elevated *Il1b* expression, whereas *Tnf* expression remained unchanged. The lack of change in *Tnf* gene expression in our study may reflect TNF-α response kinetic, which is dependent on both the time point of analysis and on the type of hepatic or non-hepatic tissue injury model used [32,33,41]. Acute post-surgical stress elevated hepatic *Tnf* expression in surgery-exposed rats, in agreement with studies that showed psychological stress primes hepatic innate immunity *via* stimulated glucocorticoid pathway [42].

However, despite our integrative approach, this study has several limitations that should be considered. Our experiments were performed in male BHR. While this model enhances translational relevance for male patients with prehypertension, it limits generalizability to females and normotensive population as sex hormones and blood pressure status are known modulators of both iron metabolism and oxidative responses. In addition, as mRNA expressions were measured in our study, the results should be interpreted with caution, since protein transcript abundance may not directly correspond to gene expression, although a positive association between *Nfe2l2* mRNA and NRF2 protein expressions has been found [43].

In conclusion, this study demonstrates that even mild surgical trauma, especially when followed by acute stress, can significantly alter iron metabolism, enhance oxidative stress, and trigger proinflammatory signalling in prehypertensive rats. These effects were associated with altered magnetic properties, suggestive of iron oxidation, and suppressed antioxidant gene expression of *Nfe2l2* and *Gpx4* which is a molecular profile favoring ferroptosis. Thus, our findings highlight the critical interplay between physical insult and acute stress in modulating hepatic iron homeostasis. Our results also suggest that modulation of redox signalling pathways, including potential therapeutic targeting of NRF2 function, may offer promising strategies to mitigate hepatic injury and improve post-surgery recovery in at-risk population.

## Figures and Tables

**Table 1:** Used primer pairs in the RT-qPCR.

Gene	Forward primer	Reverse primer	T_m_ (°C)	Amplicon size (bp)
*Nfe2l2* (NM_031789.2)	TGC CAT TAG TCA GTC GCT CTC	ACC GTG CCT TCA GTG TGC	60	102
*Ppara* (NM_013196.1)	TGA ACA AAG ACG GGA TGC TGA T	TCA AAC TTG GGT TCC ATG ATG TC	60	106
*Pparg* (NM_013124.3)	CTC ACA ATG CCA TCA GG TTT GG	GCT GGT CGA TAT CAC TGG AGA T	59	84
*Hmox1* (NM_012580.2)	AGA AGA GGC TAA GAC CGC CT	TCT GGT CTT TGT GTT CCT CTG TC	60	86
*Sod1* (NM_017050.1)	CTG AAG GCG AGC ATG GGT TC	TCC AAC ATG CCT CTC TTC ATC C	60	131
*Gpx4* (NM_017165.4)	TAA GTA CAG GGG TTG CGT GTG	CAA GGG AAG GCC AGG ATT CG	60	135
*Fpn1* (NM_133315.2)	GAC CTC ACC TAA AGA TAC TGA GCC	GAA GGG TTC TGC GAT CTG GG	59	130
*Irp1* (NM_017321.1)	ACG TCA AAA CCA GCC TGT CT	ACC ACG TCA AAC CCT AAC TGG	59	100
*Tfr1* (NM_022712.1)	GCT ATG AGG AAC CAG ACC GC	CAC TGG ACT TCG CAA CAC CA	58	78
*Dmt1* (NM_013173.2)	CTA CTT GGG TTG GCA GTG TTT G	ATC TTC GCT CAG CAG GAC TTT	60	94
*Fth1* (NM_012848.2))	GAC CTC ACC TAA AGA TAC TGA GCC	GAA GGG TTC TGC GAT CTG GG	59	130
*Hamp* (NM_053469.1)	CTA TCT CCG GCA ACA GAC GAG	TGT CTC GCT TCC TTC GCT TC	60	110
*Tnf* (NM_012675.3)	CGT CAG CCG ATT TGC CAT TTC	TGG GCT CAT ACC AGG GCT T	60	116
*Il1b* (NM_031512.2)	CAC CTC TCA AGC AGA GCA CAG	GGG TTC CAT GGT GAA GTC AAC	60	79
*Rpl10a* (NM_031065.1)	TCC ACC TGG CTG TCA ACT TC	GGC AGC AAC GAG GTT TAT TGG	60	134

Abbreviations: T_m_ melting temperature, bp – base pairs, *Nfe2l2* – Nuclear factor erythroid 2–related factor 2, *Ppara* – Peroxisome proliferator-activated receptor alpha, *Pparg* – Peroxisome proliferator-activated receptor gamma, *Hmox1* – Heme oxygenase 1, *Sod1* – Superoxide dismutase 1, *Gpx4* – Glutathione peroxidase 4, *Fpn1* – Ferroportin 1, *Irp1* – Iron regulatory protein 1 (aconitase 1), *Tfr1* – Transferrin receptor 1, *Dmt1* – Divalent metal transporter 1, *Fth1* – Ferritin heavy chain 1, *Hamp* – Hepcidin antimicrobial peptide, *Tnf* – Tumor necrosis factor alpha, *Il1b* – Interleukin 1 beta, *Rpl10a* – Ribosomal protein L10a.
